# Monitoring insect numbers and biodiversity with a vertical-beam entomological radar

**DOI:** 10.1098/rstb.2023.0117

**Published:** 2024-06-24

**Authors:** V. Alistair Drake, Zhenhua Hao, Haikou Wang

**Affiliations:** ^1^ School of Science, The University of New South Wales, Canberra, ACT 2610, Australia; ^2^ Institute for Applied Ecology, University of Canberra, Canberra, ACT 2617, Australia; ^3^ Australian Bureau of Agricultural and Resource Economics and Science, Australian Government, Canberra, ACT 2601, Australia; ^4^ Australian Plague Locust Commission, Department of Agriculture, Fisheries and Forestry, Australian Government, Canberra, ACT 2601, Australia

**Keywords:** insect, biodiversity, radar, monitoring

## Abstract

Concerns about perceived widespread declines in insect numbers have led to recognition of a requirement for long-term monitoring of insect biodiversity. Here we examine whether an existing, radar-based, insect monitoring system developed for research on insect migration could be adapted to this role. The radar detects individual larger (greater than 10 mg) insects flying at heights of 150–2550 m and estimates their size and mass. It operates automatically and almost continuously through both day and night. Accumulation of data over a ‘half-month’ (approx. 15 days) averages out weather effects and broadens the source area of the wind-borne observation sample. Insect counts are scaled or interpolated to compensate for missed observations; adjustment for variation of detectability with range and insect size is also possible. Size distributions for individual days and nights exhibit distinct peaks, representing different insect types, and Simpson and Shannon–Wiener indices of biodiversity are calculated from these. Half-month count, biomass and index statistics exhibit variations associated with the annual cycle and year to year changes that can be attributed to drought and periods of high rainfall. While species-based biodiversity measures cannot be provided, the radar's capacity to estimate insect biomass over a wide area indicates utility for tracking insect population sizes.

This article is part of the theme issue ‘Towards a toolkit for global insect biodiversity monitoring’.

## 1. Introduction

Several recent studies have reported declines in insect numbers and diversity in both natural and agricultural landscapes [1,2], leading to recognition of the need to monitor insect populations over long periods to resolve trends. Insects are an important component of almost all terrestrial ecosystems and therefore should be represented in any biodiversity assessment; however, their small size, the large number of taxa and the wide range of habitats in which they are found make monitoring challenging [3]. Practicable (and affordable) solutions are likely to involve some form of sampling, and in consequence will have limitations as all sampling methods introduce some form of bias: for example, light traps will catch only flight-capable nocturnally active species that exhibit positive phototaxis. Monitoring will need to be maintained for many years, while avoiding (or accounting for) changes in equipment or site properties that would introduce artefactual trends. Rather than proposing a novel system designed to meet these requirements, we present here an already developed insect-observation capability, based on radar technology, and examine its potential utility for assessing long-term trends in insect numbers and diversity. This ‘Insect Monitoring Radar’ (IMR) was designed to observe insects in flight at heights above approximately 150 m. It and similar units are used in research on the behaviour of insects undertaking migratory flights and on the ecological significance of the resulting population displacements (e.g. [4,5]); they have also found an operational role as an aid to insect pest forecasting [6]. Use of these radars for more general monitoring of insect numbers and biodiversity has been proposed previously [7] but not implemented. We identify both strengths and weaknesses of this radar type for this application, and suggest that radar observations, with adjustment for known biases, could form a useful component of a broader-based insect biodiversity monitoring programme (e.g. [8]).

## 2. Equipment and methods

### (a) Observing insects with radar

Radar is a remote-sensing technology that employs electromagnetic radiation at radio frequencies (broadly, 100 kHz to 100 GHz) to detect and obtain information about distant objects [9,10]. A radar transmits a radio signal, usually directed as a ‘beam’ (i.e. strong only within a narrow solid angle), and receives ‘echoes’ from any radio-reflective objects (conventionally termed ‘targets’) that the beam illuminates. Analysis of the received signals allows information about the distance of the target (its ‘range’) and its reflecting power (usually expressed as ‘radar cross section’, RCS, units m^2^ or—for insects—cm^2^) to be retrieved. The target's direction is given by the direction the radar's antenna is pointing towards. The beam may be ‘scanned’ by turning the antenna, and its elevation angle varied, to provide coverage of a wide volume of space over a period of a few minutes. Modern radars are typically ‘polarimetric’, i.e. they determine how the target affects the polarization of the echo signal as well as its intensity, and ‘coherent’, allowing them to retrieve the radial component of a target's speed and the relative strength and the phase difference of the echo signals at orthogonal polarizations. When there are multiple targets close together, as in rain or a dense insect population, the quantities retrieved are statistical analogues and extensions of those for individual targets.

Radar designs are very diverse, each optimized for its specific application: the type of target, the information about it that is required, the volume of space to be covered, the rapidity with which the information is to be obtained, whether the radar is at a fixed location or on a moving platform, the budget available, etc. [11]. Only a few designs provide useful information about insects [12]; some have been developed specifically for insect observation, and others, principally meteorological and atmospheric-science units, detect insects because they are present in the space that the radar is observing and have some similarity (as radar targets) to the objects the radar is designed to detect. The first purpose-built ‘entomological radar’ was developed in 1968 and revealed a range of spectacular insect flight phenomena [13]. This unit transmitted at a frequency of 9.4 GHz (‘X band’, 32 mm wavelength), which leads to strong reflections from larger insects such as grasshoppers and locusts, butterflies and moths, these being detected individually. Like similar units operated over the following two decades in Africa, Australia, North America and China [12], this was a scanning unit observing individual insects out to approximately 1.5 km horizontally and approximately 1 km in height. These early units were manually operated and, while effective for research, were unsuited to long-term monitoring; this was not achieved until 1990, when a radar employing a fixed (vertically directed) beam detected insects at heights from 500 to 2300 m at a site in Texas, USA [14]. Observations over two successive years revealed seasonal patterns of insect flight and their variation with height and time through both day and night [15]. RCS estimates were consistent with the targets being predominantly noctuid moths, which were known from earlier research to migrate through the region.

Since the late 1990s, vertical-beam entomological radars (IMRs and similar ‘vertical-looking radars' or VLRs) have operated continuously and unattended at sites in Australia and the United Kingdom (e.g. [6,16]) and for shorter periods in China and Japan [17,18]. Like their Texan forerunner, these radars employ pulsed transmission at X-band frequencies and are non-coherent; however, they employ a more sophisticated general design, the ‘ZLC configuration’ [12], which incorporates a very-narrow-angle conical scan and synchronous rotating linear polarization. This enables the speed, movement direction, RCS and ‘polarization pattern’ of the insect to be retrieved from the signal recorded during the insect's transit of the beam [19,20]. The polarization pattern provides information about both the alignment of the insect's body axis and its ‘shape’—though the latter is in terms of RCSs at orthogonal polarizations rather than ratios of actual body dimensions. With some target types, wing-beat frequencies can also be retrieved [21]. The RCS can be used to estimate the insect target's mass [22,23], so IMR-type radars can provide estimates of both insect numbers and biomass.

Some aspects of the ZLC configuration—its use of high-power pulses, non-coherent transmission and reception, and mechanical rotation of the antenna feed (to obtain the narrow-angle scan and rotating polarization)—now appear technologically dated. Alternatives that better reflect contemporary radar-engineering practice are becoming available, and we provide brief descriptions here of two contrasting examples. A fully polarimetric vertical-beam entomological radar employing pulse compression to provide a height resolution of 0.2 m—a huge improvement on the 7.5 m typical for an IMR—has been developed in China [24,25]. This unit operates at 16.2 GHz (18.5 mm wavelength), which potentially makes it more effective than a 9.4 GHz IMR at detecting smaller insects. In trials, it has obtained distributions of wing-beat frequencies, sizes and alignments, shown ascent and descent (at dusk and dawn respectively), and provided insect density profiles similar in quality to those from an IMR. However, it appears to have no means of determining the horizontal direction of movement, an important output for traditional IMR applications but perhaps less critical for biodiversity monitoring. The radar has been deployed in Yunnan province, China, where flights by a locust species were monitored during an outbreak [26]. Our second example, with a different set of advantages and disadvantages, is the Birdscan MR1 (Swiss Birdradar Solution AG, Winterthur, Switzerland, www.swiss-birdradar.com, accessed 7 Aug 2023), a commercially produced vertical-beam design developed principally for bird observation but that also detects insects and distinguishes them from other target types [27]. Like an IMR, this radar uses the older non-coherent technology. While it can determine the direction a target is moving, it is not optimized for insect observation and is less able to detect smaller insects or insects flying at greater heights. Its major advantages for biodiversity monitoring are that it separates birds, bats and insects, recording all three categories, and that it is available as a turnkey system with maintenance support, so it can be operated without the need for in-house radar-technology expertise. Networks of these units are already operational in Europe and delivering scientific results [28].

### (b) Hay IMRU monitoring capabilities

In this paper we will examine data from an IMR that has operated at Hay, New South Wales, Australia (34.5458° S, 144.8663° E) since 2018. Hay is located within an extensive plain where there is a mix of intensive cropping and rangeland grazing. This radar is a ‘second-generation’ unit, termed Insect Monitoring Radar—Upgraded (IMRU); it employs fully digital data acquisition and improved operating and analysis procedures, which together have increased the sample size fivefold [29]. It commenced full operation in September 2018 and the analysis presented here will cover the almost 5 year interval from then until June 2023; operating availability has ranged from 48 to 96% annually and yearly totals of fully analysed insect echoes range from 1.2 × 10^6^ to 4.1 × 10^6^ (table 1). The IMRU operates almost continuously throughout day and night, on a 15 min cycle during which data are acquired in four 3 min periods, the remaining 3 min being assigned to data preprocessing, recording to disc and performance monitoring [29]. Three of the 3 min periods in each cycle are used to observe the height range 150–1350 m with ‘short-pulse’ (0.05 µs, S) transmissions while ‘medium-pulse’ (0.25 µs, M) observations at 1200–2550 m are made during the fourth period. Breaks of duration one or two cycles are scheduled twice a day, one early in the morning and one late in the afternoon, to accommodate additional house-keeping requirements. The IMRU continues operating during rain even though its ability to detect insects is then decreased. The incidence of rain could be determined by a detector at the radar site, or from data recorded by an automatic weather station operated by the Bureau of Meteorology at Hay Airport, 3 km away, or even from the distinctive echo signals that rain produces. In this study, however, rain incidence and effects have not been considered. As rain seems generally to depress flight activity [30], this is unlikely to introduce a serious bias.

**Table 1.** Availability of Hay IMRU 2018–2023.

interval (1 July–30 June)	no. days with observations	total observing time (h)^a^	no. insects observed (×10^6^)^b^
2018–2019	179 (49.0%)	3275 (37.4%, 48.2%)	1.246 (46.3%)
2019–2020	197 (53.8%)	3521 (40.1%, 51.7%)	1.541 (57.3%)
2020–2021	342 (93.7%)	6228 (71.1%, 91.7%)	4.005 (58.3%)
2021–2022	365 (100%)	6493 (74.1%, 95.6%)	4.090 (62.0%)
2022–2023	303 (83.0%)	5197 (59.3%, 76.6%)	3.213 (63.4%)
2018–2023	1386 (75.9%)	24 714 (56.4%, 72.8%)	14.094 (59.4%)

^a^The first percentage is relative to total duration of interval (column 1), and the second is relative to aimed-for observing time, i.e. 12 min in each 15 min cycle for 93 of the 96 15 min cycles in a day, over the entire interval.

^b^Targets for which a full analysis was achieved; percentage is proportion with wing-beat frequency retrieved.

### (c) Sampling protocol and area of coverage

As the primary objective of biodiversity monitoring is to determine how population sizes and species compositions vary over multiple-year intervals, the fundamental analysis interval is 1 year. Our data are therefore organized around ‘Southern-Hemisphere Growing-Season Years' (SGY) defined to commence on 1 July and end on 30 June (i.e. in mid-winter) to conform with the annual cycle of the seasons and the modulation of ecosystem activity that this drives. Similarly, to conform with the daily cycle of insect activity, days are divided into two 12 h periods starting at 06.00 h Australian Eastern Standard Time (AEST) (day) and 18.00 h (night). As species mix and activity types differ considerably between day and night [13,31], these two periods, which are usually separated by activity minima, are analysed separately.

Individual insect species are typically only present as flight-capable adults for part of each annual cycle, so composition and numbers will vary as the season progresses, with populations of different species increasing and decreasing asynchronously—although with an overall trend of increase in spring, decrease in autumn, and a winter minimum. It seems appropriate, therefore, to provide productivity and biodiversity statistics for intervals shorter than a full year. This will enable the form of the annual cycle to be examined and allow year-to-year comparisons for each season or part of a season. Such shorter intervals seem likely to provide more specific and sensitive indicators of change than totals or averages for a full year. They should be long enough to encompass short-term weather changes, which will strongly modulate insect flight activity, but not last much longer than periods when individual insect species are active or extend over major seasonal changes in temperature or rainfall. We suggest these criteria indicate a duration of between 10 days and 2 months and have adopted the ‘half-month’ as our sampling interval. Denoted JulA, JulB (for first and second half of July), etc. these are defined as either the first 15 days of each month (14 for February) or the remaining 15 or 16 days (14 or 15 for February). Synchronizing the intervals with the familiar months assists interpretation. To eliminate effects of differing half-month lengths, insect count and mass totals are scaled to a standard 15.2 day duration (this being 1/24th of the mean annual duration of 365.24 days).

**Figure 1.**
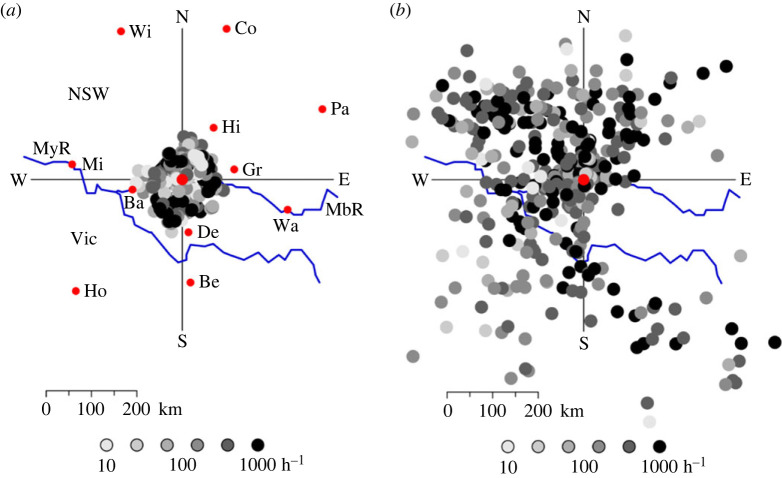
Estimated nominal locations of regions of origin for 1 h samples of insect observations by the Hay IMRU (larger red circle) during the interval 1–15 January 2022. (*a*) Day, 28 size categories, 298 1 h non-zero samples, 87 670 insect echoes; (*b*) night, 34, 390, 158 655. Locations identified by backtracking using speed and direction at time of arrival at the IMRU; flight durations of 0.5 and 1.5 h assumed for the first 2 h, then 2 h by day and the number of hours since dusk (approx. 20.00 h AEST at Hay in January; i.e. from 0.5 to 9.5 h) by night. Key to geographical features: MbR—Murrumbidge River; MyR—Murray River (and state border); NSW—New South Wales; Vic—Victoria; Ba—Balranald; Be—Benalla; Co—Cobar; De—Deniliquin; Gr—Griffith; Hi—Hillston; Ho—Horsham; Mi—Mildura; Pa—Parkes; Wa—Wagga Wagga; Wi—Wilcannia. The area is an extensive plain except along the southern and southeastern borders, where the terrain rises towards the Great Dividing Range. (Electronic supplementary material, data file 2).

An IMR observes insects at a single point as they pass overhead. However, these insects will have taken to the air some distance away, and therefore the IMR is effectively monitoring insects from a wider area. The effective sampling coverage can be estimated from observed speeds of movement and inferred flight durations. Speeds are typically in the range 5–15 m s^−1^ [6,32]. Durations for daytime flight are uncertain, and a nominal time of 2 h will be assumed. Night-time flight commences at dusk and there is little evidence of take-off occurring after that time [12,13]; therefore, insects detected by an IMR late in the night will have flown for up to approximately 10 h (depending on the latitude and season). Thus a 12 h sampling period (day or night) will include insects from all distances up to 35–100 km by day and 200–500 km by night (depending on the speed of movement, which itself depends largely on the speed of the wind). The direction of these regions of origin will also depend mainly on the direction the wind is blowing from and will typically cover a quite narrow range on a single night. However, over a longer period, changes in the wind direction will produce corresponding changes in the direction of the regions of origin of the samples. Daytime and night-time effective sampling areas are shown for a single half-month in figure 1, estimated from hourly mean track speeds and directions for insects in predominant size categories for each day or night. (Size categories are defined by peaks in the distribution of *a*_0_, a radar measure of target size; see discussion of figure 4, below.) The median distances (weighted by number of echoes) were 61 km (quartiles 50, 66 km) by day and 157 km (71, 223 km) by night. All 30° arcs received some coverage: between 11 and 47 1 h samples by day and between 4 and 67 by night.

## 3. Results

### (a) Estimation of numbers and biomass

Given the concern about insect population declines in general, an appropriate first quantity to monitor is simply the total size of all insect populations combined, without regard to their diversity. This could be defined in terms of either numbers or biomass; as insects are short-lived, the latter corresponds to the insect component of secondary biological productivity. Biomass also provides an apparently appropriate way to combine data for insects of different sizes. IMRs are very effective at detecting one component of the insect population—that for which individuals have masses of at least 10 mg and fly at heights greater than 150 m [33], and it is this component that will form the basis of any productivity or biodiversity estimates derived from IMR observations.

**Figure 2.**
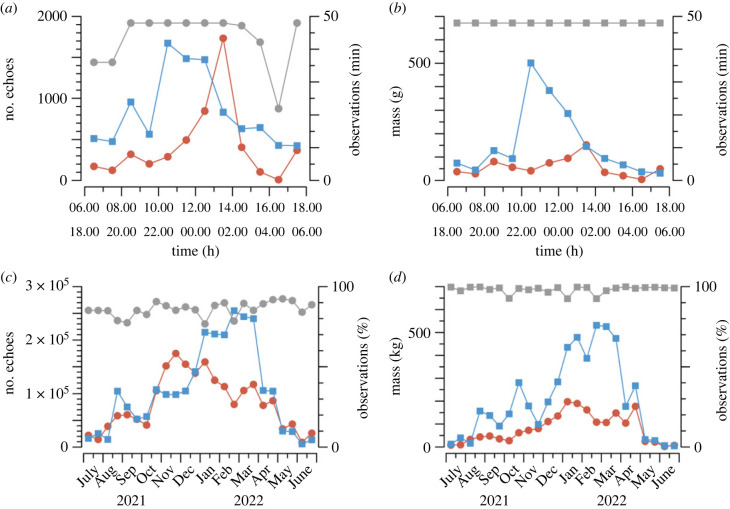
Variations of (*a,c*) insect numbers and (*b,d*) biomass for (*a,b*) the day (brown) and night (blue) of 6 January 2022 and (*c,d*) the days and nights of Southern-Hemisphere Growing-Season Year (SGY) 2021–22. Numbers and masses are totals for 1 h (48 min of observations) for (*a,b*) and for a half-month for (*c,d*), with rescaling when required (see text); interpolation was not required on 6 January 2022. The grey points indicate the duration of observations in each hour (*a*,*b*) and the proportion of possible observing duration for which observations were obtained (*c,d*). (Electronic supplementary material, data files 1 and 3.)

The diurnal pattern of insect activity for a single day and night, 6 January 2022, is shown in figure 2 in terms of both insect numbers (figure 2*a*) and biomass (figure 2*b*). The mass of each insect is estimated from its *a*_0_ value by the ‘second-order’ formula of Drake *et al*. [23] and summed to produce a total for all insects detected during each hour. Masses were limited to 1 g, which is consistent with what is known of species flying at altitude at this location [6,34]; larger masses probably arise from unrejected poor-quality echo signals. While only 4.0 and 2.4% of the daytime and night-time samples respectively were affected, the estimated total biomasses for the two 12 h periods were reduced by 40 and 13% respectively. When the duration of observations in an hour was less than the maximum of 48 min, both numbers and masses are scaled up to estimate the likely count and mass for this standard duration. Numbers and masses for the first and/or last hours of each day (i.e. 06.00, 17.00) or night (18.00, 05.00) are set to zero if there are no observations at these times, and values for any intervening hours with no observations are estimated by linear interpolation.

The annual patterns for the SGY 2021–22, with observations amalgamated into half-months, are shown in figure 2*c,d*. The hourly values for each day and each night, scaled or interpolated where necessary, are summed, and these two daily sums are totalled for each half-month interval and then scaled to the standard 15.2 day duration. Values for days or nights with no observations are estimated by linear interpolation between the nearest days with observations.

**Figure 3.**
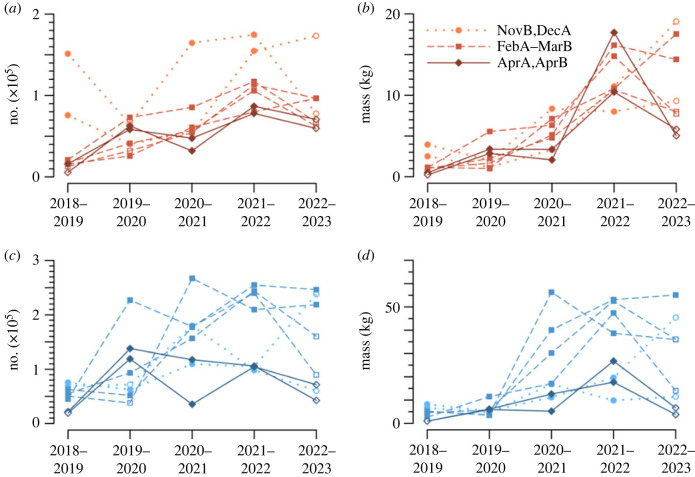
Variations over the five Southern-Hemisphere Growing-Season Years (SGYs) 2018–19 to 2022–23 of (*a,c*) insect numbers and (*b,d*) insect biomass, by (*a,b*) day (brown) and (*c,d*) night (blue), for the eight half-months NovB, DecA (dotted lines), FebA, FebB, MarA, MarB (dashed), AprA and AprB (continuous). Values that include interpolations between days are shown with open symbols. (Electronic supplementary material, data file 4.)

A critical function of a monitoring system is assessment of year-to-year changes. In figure 3, we show variations over the five SGYs for the eight half-months for which data are available in all years. Some features of insect flight activity at this site are evident by inspection: (1) more insects fly, and their biomass is greater, by night than by day; (2) year-to-year changes can be both positive and negative; (3) there were more insects, and (more clearly) greater biomass, in the three later years than in the first two, with SGY 2021–22 being strongest on both counts; (4) there is considerable variation between seasons, with early summer (NovB, DecA) having the highest insect number by day, but late summer/early autumn (FebA–MarB) strongest for the other three measures; (5) variations for consecutive or close half-months are correlated (most obviously for AprA, AprB). The larger values in the later years can be tentatively attributed to rainfall differences, as inland eastern Australia moved from drought [35] to flooding rains [36,37]. The presence of populations of Australian plague locusts, *Chortoicetes terminifera*, near Hay in 2020–21 and 2021–22 [38] will have contributed to the high night-time biomasses in the summer months of those years. Correlations for close half-months are to be expected as insect populations will mature to the adult (flight-capable) stage over an interval that is likely to extend over at least one half-month boundary.

### (b) Insect classification and biodiversity estimation

Biodiversity, by definition, implies recognition of a variety of taxa. The indices of Simpson and of Shannon (or Shannon and Wiener),

3.1 $$D=\;1-\overset{n_{s}}{\underset{i=1}{\sum }}p_{i}^{2}\;(\mathrm{Simpson})$$

and

3.2 $$H^{\prime }=\;-\overset{n_{s}}{\underset{i=1}{\sum }}p_{i}\ln p_{i}\;(\mathrm{Shannon}),$$

are two measures of diversity commonly employed in ecology [39]. Here *n*_s_ is the number of species and *p_i_* the proportion of the total population (in practice, of the total sample) of species *i*; thus, these indices incorporate both the number of species and their relative population sizes. While other measures may have advantages [40], the simple forms of these two make them well suited for an examination of whether IMR data can provide a basis for assessing biodiversity.

**Figure 4.**
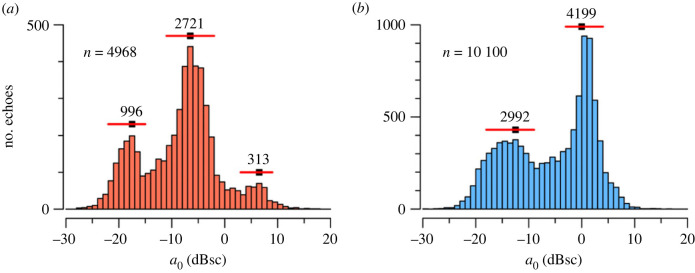
(*a,b*) Histograms of polarization-averaged radar cross-section (RCS) *a*_0_ (logarithmically transformed, decibels relative to 1 cm^2^) of insects observed by the Hay IMRU during (*a*) the day of 6 January 2022 and (*b*) the immediately following night; annotations indicate identified peaks, their widths, and their sample sizes. Masses for the marked peak points are (*a*) 22, 76 and 1200 mg, and (*b*) 38 and 240 mg, estimated from the ‘second-order’ formula of Drake *et al*. [23]. (Electronic supplementary material, data file 1.)

Radar is not able to identify individual species, for fundamental reasons: the interrogating wavelength (32 mm for the IMRU) is much longer than the size of the morphological features and patterns of coloration typically relied upon for taxonomic identification; and radio waves do not distinguish colours. The information on the ‘character’ of a target (as distinct from its height and movement) provided by an IMR for individually detected insects comprises a measure of size, two measures of ‘shape’, and often also a wing-beat frequency [34,41]. An IMR's vertical beam intercepts insects undertaking steady (non-manoeuvring) flight at a consistent aspect, i.e. ventrally if the insect is flying with its body axis horizontal—or at least consistently vertical for insects flying with non-zero (probably positive) pitch angles as some insect migrants are now known to do [42]. Moreover, the IMR's rotating polarization, which completes several cycles while the insect is transiting the beam, enables calculation of a measure of size, the polarization-averaged RCS *a*_0_, that is unaffected by the direction the insect is heading towards. This consistency of the size measure represents a significant advantage, for target characterization, of IMR-type designs over more conventional scanning configurations with slanting beams. The ability to determine size for individual insects represents an equally important advantage of IMRs over weather radars, which always receive echo from multiple insects. As *a*_0_ correlates well with insect mass [23], and retrieved values extend over at least three decades (i.e. 30 dB; [41]; figure 4), it has high potential for discriminating insect types, albeit only into broad size categories.

**Figure 5.**
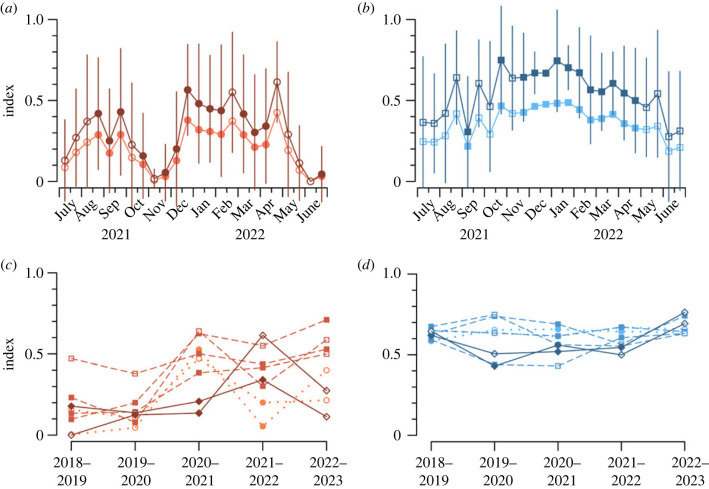
(*a,b*) Variations of means, for each half-month, of Simpson (lighter colour) and Shannon (darker) indices through Southern-Hemisphere Growing-Season Year (SGY) 2021–22; bars indicate standard deviations (for the Shannon index only). (*c,d*) Variations over the five SGYs 2018–19 to 2022–23 of the Shannon index for the eight half-months with data in all five SGYs. (*a*,*c*) Day (brown), (*b*,*d*) night (blue); lines, shades and symbols as in figure 3. (Electronic supplementary material, data file 5.)

Distributions of *a*_0_ values obtained from the Hay IMRU for individual days or nights almost always exhibit peaks that stand out from a general background distribution (figure 4*a,b*). An analogue of the Simpson and Shannon indices, in which peaks are substituted for species, can therefore be computed. Peaks have been identified objectively from single-day and single-night samples using a simple peak-and-trough algorithm [41], which also determines nominal peak widths; all echoes falling within the width are assigned to its population—i.e. no attempt has been made to determine and subtract backgrounds. An average index, and its standard deviation, are then calculated for each half-month; this is preferred to identifying peaks in combined (multiple-day or multiple-night) samples, as peaks then broaden and merge so that sensitivity to population diversity decreases. Diversity time series calculated in this way are shown in figure 5*a,b* for SGY 2021–22 and in figure 5*c,d* for the same eight single half-month sequences as in figure 3.

It is evident in figure 5*a,b* that the two indices follow each other closely, although the Simpson's value is consistently lower than the Shannon's: the former is 66 ± 5% of the latter by day and 67 ± 2% by night. The variances of the two indices are similarly related: the Simpson's standard deviation is 66 ± 5% of the Shannon's by day and 65 ± 10% by night. For simplicity, hereafter we will consider only the Shannon index. Values are somewhat higher by night than by day, and in both cases the standard deviations are large, sometimes larger than the index itself, indicating high day-to-day variation within a half-month. Nevertheless, some features of the annual cycle are evident. In both the day and night series, there is a maximum in mid-SGY, when temperatures are higher and samples are larger, and a minimum at the beginning and end of the year, i.e. in winter. In the daytime series there is a strong dip in spring, reaching a minimum with almost no diversity in NovA. Inspection of daytime *a*_0_ histograms for this interval revealed that a strong and quite narrow peak near −15 dBsc (approx. 30 mg) was the only significant feature of the size distribution on almost every occasion; at night, and at other times of the year, two or three peaks were commonly present.

The index value is strongly correlated with the number of peaks (Pearson correlation coefficient 0.96 by day, 0.94 by night for the SGY 2012–22 sample). As a minimum number of echoes (approx. 200) is needed to identify even a single peak, lower index values in the cooler months (figure 5*a,b*) might be due simply to fewer insects being detected then (figure 3*a,b*). However, the correlation between the index and the number of insects detected (log_10_-transformed) is much lower (0.32 by day, 0.39 by night) and hardly different from the correlation between the number of insects (log_10_-transformed) and the number of peaks (0.31, 0.37); we therefore conclude that the index values are not an artefact of sample size. The values obtained (maximum 1.33 both by day and by night), which are low in comparison with those typical of conventional ecological surveys (1.5–4.5 [43]), reflect the small number of peaks resolvable in the *a*_0_ distributions. The most resolved was four, for both day and night, which leads to a theoretical maximum Shannon index value of ln4 ≈ 1.39. The small number of peaks can in turn be attributed to their widths (figure 4), which may be due either to size variation among the insects forming the peak sample, or to inaccuracies arising in the radar measurements and analyses, or to a combination of these. The large standard deviations of figure 5*a,b* can be understood as a consequence of variation in the number of peaks about a small mean, combined with the strong correlation between the number of peaks and the index value. Over the 5 year interval (figure 5*c,d*), there is more variation, both from year to year and between half-months of the same year, by day than by night.

## 4. Discussion

The aim of this study was to determine how well an existing insect-observation system, based on a vertical-beam radar developed for research on insect migration, would function as a system for assessing changes in insect numbers and biodiversity. Because radar cannot identify species, this technology is unable to provide measures of diversity comparable to those conventionally employed in ecology [44]. However, we propose that it might play a useful ancillary role, which we have explored here. Our investigation shows that radar has strengths as well as weaknesses as a tool for monitoring insect populations. Its capacity to assess insect productivity over a wide area and continuously could be valuable both in itself (as a direct measure of insect population changes) and as a baseline enabling informed interpretation of higher-order biodiversity measures obtained by other, more species-focused, techniques.

Like other automated systems, radar has the capacity to observe continuously with only modest operating costs (electricity, telecommunications, maintenance), though purchase/lease and installation costs will be significant. Continuous operation avoids the risk of missing important or unexpected events and maximizes sample size. Unlike conventional insect sampling, the data collection is not followed by a potentially slow and costly process of manual sorting and identification of catches. Thus radar, along with optoelectronic (e.g. [45,46]) and acoustic [47] insect monitoring systems, meets a primary requirement of any biodiversity monitoring programme: the capacity for operation to be maintained over decadal timescales. Of course, this will require appropriate technical support, not just for routine maintenance but to adapt to changing technologies as outdated components become unavailable and better-performing and lower-cost alternatives need to be substituted—as already experienced with IMRs [29].

All monitoring systems inevitably have some downtime due to equipment failures, inclement weather, and essential maintenance and housekeeping. Any data loss will reduce confidence in the accuracy of the system's data products, but we show that the precise record-keeping provided by an automated system makes it possible to fill shorter gaps (hours, days) by rescaling and interpolation to produce normalized measures of activity for standard intervals: first 12 h daytime and night-time periods and then 15.2 day half-months. More sophisticated methods incorporating typical diurnal variations could be implemented for occasions when several hours of observations are lost—when interpolation would miss significant activity peaks or troughs. These adjustments reduce potential biases in time series from a single radar (such as the annual cycles of figures 2*c,d* and 5*a,b*) and in comparisons between observations from identical radars at different sites.

In this study, for clarity and simplicity, we have not considered biases arising from a radar's reduced ability to detect smaller targets, targets at greater heights, and targets passing at greater distances from the beam axis. Methods to compensate for these have been described elsewhere [22,48] and are employed in other applications of radars of the IMR type (e.g. [16]). These adjustments are needed to compare numbers and biomasses from radars with different design parameters (e.g. transmission power, beam width). Even for time series from a single radar, accounting for these effects will eliminate biases arising from changes in the heights at which the insects are predominantly flying. A particular difficulty arises for smaller insects, which may be flying in significant numbers above the maximum height at which they can be detected, and for which no information is therefore available. Eliminating smaller insects from the samples, even when they are detected at lower heights, may be the best course in these circumstances. While these normalization processes are somewhat involved, they meet a second requirement of a practical monitoring programme: quantitative comparability of values obtained from equipment that may differ between sites or perform differently following maintenance or renewal.

A third requirement of a monitoring programme is that the outputs it produces are representative of a well-defined and ecologically meaningful quantity. Here the wide effective coverage—of order 100 km—of an IMR (figure 1) and continuous operation ensure a well-mixed sample drawn from a wide area that includes species active by night as well as by day. However, only insects that take to the air, and attain heights of 150 m or more, are included. In the rangelands and crops around Hay, where summers are hot and generally dry, the proportion of the total insect population that becomes airborne when it reaches the adult stage may be high, but in other habitats, especially wet forests, many more insects may remain within the canopy than venture above it. Habitats will often vary over distances of approximately 100 km, leading to potential biases if prevailing winds change from one season to another or there are directions from which winds rarely blow. Making unbiased comparisons of biodiversity at different sites with different habitat types appears intrinsically difficult, irrespective of the monitoring technique employed.

The measure of biodiversity considered here, the Shannon index based on peaks in the *a*_0_ distribution, is practicable to compute and in most instances the peaks are well separated and clearly represent different insect types. This measure makes no use of the targets' shape or wing-beat values. It was expected that incorporating these would increase the number of distinguishable ‘radar taxa’ and thus improve sensitivity to differences in diversity. However, exploratory investigations did not support this. For example, the objective four-dimensional peak-finding algorithm of Drake [41] confirmed only a minority of the peaks identified from *a*_0_ alone, leading to a lower index value with reduced variation. Simply assigning insects to cells in a three-dimensional (*a*_0_, the shape parameter *p*, and wing-beat frequency) matrix produced only blurred patterns, with the *p* and wing-beat dimensions providing little discriminatory power and the fixed boundaries often dividing and blurring the *a*_0_ peaks. These disappointing results may be attributed to *p* varying with *a*_0_ [23] and wing-beat frequency distributions often showing only one well defined peak, so neither of these dimensions reveals additional insect types. A further disadvantage of using wing-beat frequency is that it is not retrieved for all detected insects, so sample sizes are reduced—and probably in a taxonomically biased way, as different body sizes, morphologies, and flight modes will affect how strongly RCS is modulated by wing movement. Further exploration of the use of shape and wing-beat data is warranted, but at this stage relying on *a*_0_ values alone, and basing the biodiversity measure on objectively identified peaks in the *a*_0_ distribution, appears most satisfactory. Some optoelectronic techniques can obtain additional target characters (e.g. pigmentation, wing thickness [49,50]) and may therefore provide more sensitive measures of biodiversity, though they will be for a very different and spatially restricted sample.

The 5 year productivity series presented here (figure 2*c,d*) is clearly not long enough to determine if there is a trend of decreasing or increasing insect numbers. Rather, there is considerable year-to-year variability, and over this interval an increase which can be attributed to rainfall variability that is in turn driven mainly by the El Niño–Southern Oscillation teleconnection over the Pacific Ocean [51]. Given such variability, it will be many years before evidence for a gradual trend becomes compelling. Even with a catastrophic drop in numbers, it would need to be shown that this was not simply an effect of drought—as in the first two years of our series. These are difficulties all monitoring programmes will face, and the normalized biomass estimates from an IMR, with their wide effective spatial coverage and large sample sizes, appear well suited to monitoring this simple but important component of insect biodiversity.

## Data Availability

Datasets supporting this article, and analysis software, are provided in the electronic supplementary material [52].
